# The Role of High-Density Lipoprotein in COVID-19

**DOI:** 10.3389/fphar.2021.720283

**Published:** 2021-07-16

**Authors:** Guyi Wang, Jiayi Deng, Jinxiu Li, Chenfang Wu, Haiyun Dong, Shangjie Wu, Yanjun Zhong

**Affiliations:** ^1^ Department of Critical Care Medicine, The Second Xiangya Hospital, Central South University, Changsha, China; ^2^ Department of Respiratory, The Second Xiangya Hospital, Central South University, Changsha, China

**Keywords:** COVID-19, SARS-CoV-2, lipoproteins, HDL, angiotensin-converting enzyme 2

## Abstract

The current Coronavirus disease 2019 (COVID-19) pandemic has become a global challenge. Managing a large number of acutely ill patients in a short time, whilst reducing the fatality rate and dealing with complications, brings unique difficulties. The most striking pathophysiological features of patients with severe COVID-19 are dysregulated immune responses and abnormal coagulation function, which can result in multiple-organ failure and death. Normally metabolized high-density lipoprotein (HDL) performs several functions, including reverse cholesterol transport, direct binding to lipopolysaccharide (LPS) to neutralize LPS activity, regulation of inflammatory response, anti-thrombotic effects, antioxidant, and anti-apoptotic properties. Clinical data shows that significantly decreased HDL levels in patients with COVID-19 are correlated with both disease severity and mortality. However, the role of HDL in COVID-19 and its specific mechanism remain unclear. In this analysis, we review current evidence mainly in the following areas: firstly, the pathophysiological characteristics of COVID-19, secondly, the pleiotropic properties of HDL, thirdly, the changes and clinical significance of HDL in COVID-19, and fourthly the prospect of HDL-targeting therapy in COVID-19 to clarify the role of HDL in the pathogenesis of COVID-19 and discuss the potential of HDL therapy in COVID-19.

## Introduction

Coronavirus disease 2019 (COVID-19), caused by severe acute respiratory syndrome coronavirus 2 (SARS-CoV-2), outbroke in Wuhan in late 2019 (Guan W.-J. et al., 2020; Wang et al., 2020a; Huang C. et al., 2020; Hui et al., 2020; Lu et al., 2020). It has since spread worldwide (Albarello et al., 2020; Giunta et al., 2020; Young et al., 2020). By June 30, 2021, more than 180 million people have been infected with SARS-CoV-2, and nearly four million have died globally (WHO, 2021a). The COVID-19 pandemic has become a significant burden on global healthcare systems. Patients with COVID-19 with underlying metabolic dysfunction, such as type 2 diabetes and non-alcoholic fatty liver disease, have a higher risk of poor outcomes (Guan W.-j. et al., 2020; Mahamid et al., 2020; Ji et al., 2021). A decline in total cholesterol, high-density lipoprotein (HDL), and low-density lipoprotein (LDL) levels in patients with COVID-19 has been observed in several studies, including our previous research (Wei et al., 2020a; Wang et al., 2020b). Our data also shows that among the several lipids named above, only HDL was associated with the severity of COVID-19 (Wang G. et al., 2020). In this review, we aim to analyze the available evidence about how HDL dysfunction is associated with infection, including a focus on COVID-19.

## SARS-CoV-2

SARS-CoV-2 is a positive-sense, single-stranded RNA virus, surrounded by an envelope (Han et al., 2020; Kočar et al., 2021). SARS-CoV-2 is reported to share 79.6% homology with SARS-CoV (Zhou F. et al., 2020). The highly pathogenic CoVs, including Middle East Respiratory Syndrome (MERS) CoV, SARS-CoV-1, and SARS-CoV-2, mainly invade the lower respiratory tract through the upper respiratory tract and result in fatal pneumonia (Han et al., 2020).

SARS-CoV-2 entry into susceptible host tissue cells depends on the host cell angiotensin-converting-enzyme 2 (ACE2) receptor via the spike (S) protein, followed by S protein cleaving and membrane fusion (Chambers et al., 2020). ACE2 is widely expressed in human tissues, including in lung alveolar epithelial cells, small intestinal epithelial cells, vascular endothelial cells and smooth muscle cells within the lung, kidney, intestines, and other organs (Kočar et al., 2021).

## Pathophysiological Characteristics of COVID-19

COVID-19 causes significant infection-related morbidity and mortality. There have been about 33 million positive cases and nearly 600 thousand deaths in America (WHO, 2021b), while in China, there have been about 118 thousand positive cases and about five thousand deaths (WHO, 2021c). A recent meta-analysis of 212 studies from 11 countries/regions involving 281,461 individuals showed about 22.9% of patients with COVID-19 had severe disease and 5.6% patients die (Li J. et al., 2021). The most striking pathophysiological feature of patients with severe COVID-19 is a dysregulated immune response, characterized by lymphopenia and a cytokine storm, which results in acute respiratory distress syndrome, hepatic dysfunction, multiple-organ failure, and ultimately death. Abnormal coagulation function is also a prominent feature in severe COVID-19 cases (Beltrán-García et al., 2020; José et al., 2020; Song et al., 2020; Zafer et al., 2021).

### Dysregulated Immune Responses

SARS-CoV-2 may activate both innate and adaptive immune responses in patients, including lymphopenia, cytokine release syndrome, and abnormal activation of macrophages and their complement system (Jamal et al., 2021). Lymphopenia, involving a drastic reduction in T-cells and B cells (Qin et al., 2020a; Tan et al., 2020; Xu et al., 2020), is a common feature in patients with severe COVID-19. This is possibly triggered by SARS-CoV-2-induced activation of apoptosis in lymphocytes (Xiong et al., 2020).

Patients with COVID-19 have also shown monocyte/macrophages morphological and physiological changes. These monocytes were characterized by mixed M1/M2 polarization, relatively elevated CD80^+^ and CD206^+^ expression, and higher secretion of interleukin (IL)-6, IL-10, and tumor necrosis factor (TNF)-α (Zhang D. et al., 2021). Macrophages infiltrated into the lungs of patients with COVID-19 were mostly type 1 (Yao et al., 2020). Monocytes obtained from patients with COVID-19 were shown to express ACE2 receptors, suggesting SARS-CoV-2 may directly infect and affect monocytes and macrophages in COVID-19 (Zhang Y. et al., 2021). Additionally, cytokine storms were common in patients with severe COVID-19. Patients exhibited increased cytokine secretion, particularly IL-2, IL-4, IL-6, IL-10, TNF-α, and interferon (IFN)-γ (Qin et al., 2020b). The possible causes of this cytokine release syndrome could be a dysregulated immune response incapable of controlling the production of excessive amounts of cytokines and chemokine.

The complement system was also considered to play a pivotal role in COVID-19. A recent study showed that complement components of the classical (C1q, C4d) and alternative (Factor H, C3d) pathways were deposited in the lungs of people with COVID-19, indicating the activation of complement system in COVID-19 (Satyam et al., 2021). Early clinical reports indicates that C3 inhibition therapy holds potential anti-inflammatory properties in COVID-19 (Mastaglio et al., 2020; Satyam and Tsokos, 2020) and anti-complement C5 therapy in patients with severe COVID-19 lead to a drop in inflammatory markers and a successful recovery (Diurno et al., 2020; Satyam and Tsokos, 2020).

### Abnormal Coagulation

Abnormal coagulation function is also a prominent feature in severe COVID-19 cases. Severe COVID-19 was associated with widespread activation of the coagulation system, corroborated by elevated activated partial thromboplastin time (APTT) and prothrombin time (PT) along with markedly elevated D-dimer levels (Tang et al., 2020; Zhou P. et al., 2020). Severe endothelial injury and widespread thrombosis with microangiopathy are evident in lungs from patients with COVID-19 (Ackermann et al., 2020). Possible causes include a direct attack by the virus on the endothelial cells via ACE-2 receptors (Ackermann et al., 2020), and cytokine storms such as TNF and IL-6, which are potent activators of the tissue factor (TF)-dependent coagulation cascade (Tijburg et al., 1991; Kerr et al., 2001).

## Composition, Metabolism and Function of HDL

HDL is a type of lipoprotein with an extremely heterogeneous composition, density, and particle size, containing cholesterol, phospholipids, triglycerides, and apolipoproteins. It was first isolated from blood in the 1960s by ultracentrifugation. Among all types of human plasma lipoproteins, HDL, mainly synthesized in the liver and small intestine, has the highest density and smallest volume in the circulatory system. Apolipoprotein A-I (ApoA-I) is the main structural protein component of HDL, and other protein components such as serum amyloid A (SAA), lecithin cholesterol acyltransferase (LCAT), paraoxonase-1 (PON-1) and cholesterol ester transfer protein (CETP) also participate in the metabolic process of HDL (Gordon et al., 1989; Ginsberg, 1998; Tosheska Trajkovska and Topuzovska, 2017).

### Reverse Cholesterol Transport

Normally metabolized HDL has various functions. The most important and well characterized function is the regulation of reverse cholesterol transport. During the formation and maturation of HDL, its main functional protein, ApoA-I, continuously binds to free cholesterol in tissue cells, and is then transported to the liver. Thus, cholesterol is excreted from the body’s tissue cells through a series of transport and transformation processes, which reduces the cholesterol level in the body and delays the occurrence and progression of coronary heart disease (Gordon et al., 1989; Rader, 2003; Tosheska Trajkovska and Topuzovska, 2017).

### Direct Binding to Lipopolysaccharide and Neutralizing LPS Activity

LPS is the chief component of the outer membrane of Gram-negative bacteria. Numerous studies have found that HDL prevents systemic endotoxemia by binding and neutralizing LPS (Parker et al., 1995), which is considered to be the main mechanism of HDL’s antimicrobial effect (Ulevitch et al., 1979; Freudenberg et al., 1980). Early studies have shown that HDL can prevent the activation of peripheral blood monocytes and macrophages by LPS, and reduce the synthesis and secretion of inflammatory cytokines such as TNF-α and IL-1β (Levine et al., 1993). *In vivo* studies have shown that the initiation of intravenous infusion of recombinant HDL prior to induction of endotoxemia in healthy volunteers significantly reduced TNF, IL-6, and IL-8 levels, as well as reducing endotoxin-induced clinical symptoms and leukocyte activation (Pajkrt et al., 1996). A recent study showed that, compared with normal mice, ApoA-I (the main component of HDL) knockout mice showed increased production of inflammatory cytokines, decreased ability to neutralize and clear LPS, and reduced survival (Guo et al., 2013). In addition to binding and neutralizing LPS, HDL also promotes LPS clearance, mainly binding with SR-B1 and mediating LPS intake. It has been reported that in LPS-induced endotoxemia and cecal ligation and puncture (CLP) sepsis models *in vitro*, SR-B1 gene deletion mice showed decreased endotoxin clearance (Cai et al., 2008; Guo et al., 2009).

### Regulation of Inflammatory Response

HDL may also be a key regulator of inflammatory response. *In-vitro* cell experiments show that HDL inhibits a subset of LPS-stimulated macrophage genes that regulate the type I interferon response via microarray analysis (Suzuki et al., 2010). HDL also down-regulates the expression of Toll-like receptor (TLR)-induced pro-inflammatory cytokines through the transcriptional regulator activating transcription factor 3 (ATF3) (De Nardo et al., 2014). Transgenic mice with 2-fold-elevated plasma HDL levels had lower plasma cytokine levels, and improved survival rates in an endotoxemia mouse model (Levine et al., 1993).

### Anti-Thrombotic Effects

HDL can act as a regulator of platelet and coagulation responses in a variety of ways. Numerous epidemiological studies have established an inverse correlation between HDL levels and the risk of thrombosis (Sharrett et al., 2001; Deguchi et al., 2005; Lüscher et al., 2014), and many studies have explored the mechanisms involved. HDL stimulates NO and prostacyclin production in endothelial cells which are both inhibitors of platelet activation (Van Sickle et al., 1986; Yuhanna et al., 2001; Calabresi et al., 2003). Endothelial cells express TF after thrombin-induction in acute coronary syndromes, and HDL presents an atheroprotective effect by inhibiting thrombin-induced human endothelial TF expression (Viswambharan et al., 2004). HDL, mainly ApoA-I, also protects endothelial cells against oxidized LDL (oxLDL) and prevents its apoptosis (Suc et al., 1997). Additionally, purified HDL enhances inactivation of coagulation factor Va by activated protein C (APC) and protein S (Griffin et al., 1999). ApoA-I also neutralizes the procoagulant properties of anionic phospholipids, and incorporation of ApoA-I in anionic vesicles prevents the formation of the prothrombinase complex (Oslakovic et al., 2009; Oslakovic et al., 2010).

### Antioxidant and Anti-Apoptotic Properties

HDL can prevent intracellular reactive oxygen species (ROS) production, triggered by oxLDL or H_2_O_2_, thereby inhibiting the subsequent proteasome activation, and NF-kappa B activation (Robbesyn et al., 2003). HDL exerts a protective effect against oxidative damage induced by copper ions (Ferretti et al., 2003). Additionally, PON-1 is an HDL-associated esterase, which protects lipoproteins against oxidation. It is demonstrated that PON-1-deficient mice were susceptible to oxidative stress and HDL isolated from these mice were unable to prevent LDL oxidation (Shih et al., 1998).

HDL was shown to have the capacity to inhibit apoptosis of endothelial cells induced by oxLDL (Suc et al., 1997). HDL also prevented caspase-3 and caspase-9 activation, as well as apoptotic alterations of the plasma membrane (Nofer et al., 2001). In addition, HDL reduced cardiomyocyte apoptosis in a mouse model of myocardial ischemia/reperfusion (Theilmeier et al., 2006).

## HDL Changes During Human Infection

### Changes in Levels or Functions of HDL

Levels and functions of HDL changed significantly in patients infected with different pathogens. Multiple studies show HDL decreased in many infections, including sepsis, nosocomial infections, dengue, Helicobacter pylori infection, and HIV infection (Canturk et al., 2002; van Leeuwen et al., 2003; Chien et al., 2005; Rose et al., 2006; Jia et al., 2009; Aragonès et al., 2010; Baker et al., 2010; Zou et al., 2016; Cirstea et al., 2017; Tanaka et al., 2017; Barrientos-Arenas et al., 2018). The explanations includes decreased HDL synthesis, over-consumption of HDL particles, or HDL redistribution from intravascular to extravascular space (Pirillo et al., 2015; Tanaka et al., 2020a; Cao et al., 2020). Infection not only leads to a decrease in HDL levels, but also affects its function. HDL from HIV+ individuals has reduced antioxidant function (Angelovich et al., 2017), modified HDL metabolism, and reduced functionality of reverse cholesterol transport (Rose et al., 2008). In mice models, HDL loses its anti-inflammatory properties after acute influenza infection (Van Lenten et al., 2001).

### Relationship Between HDL, Susceptibility of Infection and Outcome

Low serum HDL levels seem to be associated with a higher risk of infectious diseases, including sepsis (Shor et al., 2008; Grion et al., 2010), nosocomial infection after surgery (Delgado-Rodriguez et al., 1997; Canturk et al., 2002), and in-hospital infection in patients with acute ischemic stroke (Rodríguez-Sanz et al., 2013). Furthermore, a prospective population-based cohort study involving more than 100,000 patients showed a U-shaped association of HDL with the risk of infectious disease, and that both high and low levels of HDL were related with a high risk of infection (Madsen et al., 2018). In addition, mortality rates, intensive care unit (ICU) stay, and length of hospital stay, all increased among septic patients with lower levels of HDL or ApoA-I (Chien et al., 2005; Montero-Chacón et al., 2020). Low HDL level at admission was also associated with severe sepsis (Grion et al., 2010). Lower HDL may herald a bad outcome, while higher levels of HDL seem to have a protective effect toward infection.

### Therapeutic Strategy Targeting HDL in Infection

Due to the important role of HDL in infection, therapeutic strategy targeting HDL is considered as a possible new approach to the treatment of infection. Reconstituted HDL was shown to reduce inflammation and bacterial burden, attenuate organ injury and improve survival in experimental septic models (Levine et al., 1993; McDonald et al., 2003; Tanaka et al., 2020b; Tanaka et al., 2020c). Trypanosome lytic factor, as a minor subfraction of HDL, ameliorates Leishmania infection, possibly due to the ability to selectively damage pathogens in phagolysosomes (Samanovic et al., 2009). CETP is a key regulator of HDL levels. Its gain-of-function variant was significantly associated with an increased risk of mortality in sepsis (Trinder et al., 2019). CETP inhibitor Anacetrapib preserved levels of HDL and ApoA-I and increased the survival rate in CLP sepsis models (Trinder et al., 2021).

## Association Between HDL and COVID-19

### Alteration of HDL Level in Patients With COVID-19

A large number of studies have shown a close correlation of HDL with COVID-19, which were summarized in Table 1. The serum HDL level in patients with COVID-19 was lower than that in healthy controls (Huang et al., 2021). A genome-wide association study (GWAS) summary analysis of 7362 COVID-19 participants from the United Kingdom Biobank, showed that individuals with a lower level of HDL were more vulnerable to SARS-CoV-2 infection (Zhang D. et al., 2021). A clinical observational study also found that lower HDL levels were related to a higher risk of SARS-CoV-2 infection (Aung et al., 2020), while higher HDL levels were associated with a lower risk of SARS-CoV-2 infection (Ho et al., 2020).

**TABLE 1:** Changes in HDL levels in patients with COVID-19.

Author	Country	Number of patients	Time point	Comparison of HDL levels
Wang (Wang et al. (2020a)	China	228	Within 24 h after admission	COVID-19 patients vs healthy control: median, 0.78 vs 1.37 mmol/L, *p* < 0.001
				Severe vs non-severe patients: median, 0.69 vs 0.79 mmol/L, *p* = 0.032
Huang (Huang et al. (2021)	China	218	The 1st day of admission	COVID-19 patients vs healthy control: mean, 1.02 vs 1.52 mmol/L, *p* < 0.05
				Severe vs non-severe patients: mean, 0.83 vs 1.15 mmol/L, *p* < 0.05
Zhang (Zhang B. et al. (2020)	China	74	Not known	Severe vs non-severe patients: median, 0.92 vs 1.08 mmol/L, *p* = 0.021
Xie (Xie et al. (2020)	China	62	Not known	Severe vs non-severe patients with CVD: median, 1.1 vs 1.4 mmol/L
				Severe vs non-severe patients without CVD: median, 1.1 vs 1.3 mmol/L
Hu (Hu et al. (2020)	China	114	On admission	COVID-19 patients vs healthy control: mean, 1.08 vs 1.27 mmol/L, *p* < 0.001
				Severe vs non-severe patients: median, 1.01 vs 1.21 mmol/L, *p* < 0.001
Wei (Wei et al. (2020b)	China	597	Not known	Mild vs severe vs critical patients: median, 50 vs 50 vs 36 mg/dL, *p* < 0.05
Tanaka (Tanaka et al. (2020a)	France	48	Upon ICU admission	Alive vs dead patients on day 28: median, 0.6 vs 0.5 mmol/L, *p* = 0.036
Huang (Huang W. et al. (2020)	China	2,623	At admission	Critical vs non-critical patients: median, 0.86 vs 0.95 mmol/L, *p* < 0.001
Sun (Sun et al. (2020)	China	99	Within 24 h of admission	Mild vs severe: median, 1.18 vs 0.94, *p* < 0.001
Ouyang (Ouyang et al. (2020)	China	107	Last result	Survivors vs non-survivors: average, 1.07 vs 0.79 mmol/L, *p* = 0.006
Li (Li Y. et al. (2021)	China	424	Not known	Survivors vs non-survivors: median, 0.9 vs 0.8, *p* = 0.001
Turgay (Turgay Yıldırım and Kaya (2021)	Turkey	139	At admission	Survivors vs non-survivors: median, 44.0 vs 28.5 mg/dL, *p* < 0.001

Abbreviations: HDL, high-density lipoprotein; COVID-19, Coronavirus disease 2019.

### Changes of HDL Function in Patients With COVID-19

In addition to HDL levels, the composition and functions of HDL in COVID-19 were also changed. ApoA-I and PON-1 were less abundant in patients with COVID-19, whereas, using proteomic analyses, SAA and alpha-1 antitrypsin were found to be higher (Begue et al., 2021). HDL from patients with COVID-19 showed less protection in TNF-α treated endothelial cells (Begue et al., 2021). Generally, patients with diabetes and elderly patients showed a higher extent of glycation (Kawasaki et al., 2002; Park and Cho, 2011). Glycated HDL showed much lower antivirus activity against SARS-CoV-2 than that of native HDL, which may explain why older patients and patients with underlying conditions such as diabetes are more likely to develop severe illness and death in COVID-19 (Cho et al., 2021).

### Relationship of HDL With the Outcomes of Patients With COVID-19

Additionally, HDL or ApoA-I levels were significantly lower in severe, critically ill and mortality groups compared to patients with mild COVID-19 (Wang et al., 2020b; Huang C. et al., 2020; Ouyang et al., 2020; Xie et al., 2020; Zhang Q. et al., 2020; Hilser et al., 2021; Li J. et al., 2021; Turgay Yıldırım and Kaya, 2021). This suggests that HDL is associated with COVID-19 severity and risk of death (Tanaka et al., 2020b; Hu et al., 2020; Wang et al., 2020; Wei X. et al., 2020; Zhang B. et al., 2020). During ICU hospitalization in patients with COVID-19, in cases of bacterial superinfection, low HDL concentrations were also found to be correlated with higher mortality (Tanaka et al., 2020c). Significantly, in patients with severe COVID-19, a gradual increase of HDL levels during hospitalization could suggest a path to gradual recovery (Qin et al., 2020a). Moreover, HDL levels influenced the virus shedding duration in patients with COVID-19 (Ding et al., 2020) and may predict the risk of hospitalization for COVID-19 (Hamer et al., 2020; Lassale et al., 2021). This data strongly suggests that high HDL levels might be beneficial in patients with COVID-19 through its antiviral activity.

### The Possible Mechanism of HDL Action in COVID-19

Lipid metabolism plays an essential role during SARS-CoV-2 infection. Cholesterol is widely shown to interact with SARS-CoV-2 S protein (Kočar et al., 2021). The accumulation of lipids was observed in SARS-CoV-2 infected cells, both *in vitro* and in the lungs of patients of COVID-19 (Nardacci et al., 2021). In a cell experiment *in vitro*, HDL showed an obvious antiviral effect on SARS-CoV-2 via cytopathic effect (CPE) and inhibition activity tests (Cho et al., 2021). Although HDL is believed to play a protective role in infection, some studies have come to the opposite conclusion. Several studies showed HDL facilitated SARS-CoV-2 infection. It was found that HDL significantly increased cell-surface SARS-CoV-2-S binding, viral entry and replication *in vitro* through SR-B1. Blockade of the cholesterol-binding site on SARS-CoV-2 or treatment with HDL SR-B1 antagonists, inhibits HDL-enhanced SARS-CoV-2 infection (Wei et al., 2020a). Another study showed that pretreatment of 293T cells with an HDL antagonist, in the presence of HDL, strongly inhibited the entry of SARS-CoV-2 into host cells (Wei et al., 2020). It suggested that the down-regulation of HDL levels in patients with COVID-19 may be due to HDL consumption during viral invasion, and HDL or SR-B1 could be treatment targets for COVID-19. However, future studies will need to explore the molecular nature of the interaction between HDL and SARS-CoV-2.

Additionally, clinical data showed influencing the inflammatory response may be one of the mechanisms of HDL involvement in the pathophysiology of COVID-19. Severe COVID-19 is considered to be a sepsis induced by SARS-CoV-2 (Colantuoni et al., 2020; Lin, 2020; Shenoy, 2020), which is characterized by excessive inflammation and multiple-organ failure. It is reported that a marked increase in inflammatory factors occurs in COVID-19, including C-reactive protein (CRP), IL-6, TNF-α, etc. (Song et al., 2020; Zafer et al., 2021). ApoA-1 and HDL levels were shown to be negatively correlated with CRP and IL-6 levels in patients with COVID-19 (Hu et al., 2020; Sun et al., 2020), suggesting that the increased inflammatory response related to reduced HDL levels is one of the pathogenic mechanisms of COVID-19.

Moreover, apoptosis, oxidative stress and abnormal blood coagulation are all involved in the pathophysiological process of COVID-19 (Tang et al., 2020; Cizmecioglu et al., 2021; Mehri et al., 2021), and multiple studies demonstrated HDL had anti-thrombotic, anti-apoptotic and anti-oxidative effects (Tanaka et al., 2020a), which offers a possibility that HDL may also regulate these pathways in COVID-19. However, further research is needed to confirm these conclusions.

### Therapeutic Strategy of COVID-19 Through Targeting HDL

Until now effective therapeutic interventions for COVID-19 are limited. Drug repurposing could identify potential treatments in a short time, which has become an important approach to explore therapeutic agents for COVID-19 (Kost-Alimova et al., 2020). As many studies have found that HDL is closely linked to COVID-19, some related randomized controlled trial (RCT) studies remain ongoing (Table 2). Omega-3 polyunsaturated fatty acids (PUFAs) improve lipid metabolism by reducing triglyceride and increasing HDL (Yanai et al., 2018), which enhance patient’s immune function and reduce inflammatory responses (Ni et al., 2020; Sorokin et al., 2020). Subsequently, as it is considered to have a positive role in the treatment of COVID-19, it has become the most studied lipid-regulating drug in COVID-19. Statins, including Atorvastatin, Rosuvastatin, and Simvastatin also showed HDL-increasing capacity (Jones et al., 2003; Miller et al., 2004; Rosenson, 2005; Sasaki et al., 2013), and are one of the current research hotspots of COVID-19. Moreover, RCT studies on the effects of two classic HDL-increasing drugs, CETP inhibitor Dalcetrapib and Fenofibrate, in patients with COVID-19, are also underway. Other HDL-raising pharmacological compounds such as LCAT, have been also considered as potential therapies for COVID-19 (Sorokin et al., 2020).

**TABLE 2:** Clinical studies about drugs that affect HDL on patients with COVID-19.

Therapy	ClinicalTrials.gov Identifier	Mechanism of action	Effect on HDL
**Dalcetrapib**	NCT04676867	CETP inhibitor	Raise HDL levels
**Omega3-FA**	NCT04658433	Multifactorial	Raise HDL levels or affect HDL metabolism
	NCT04553705 NCT04483271		
**Fenofibrate**	NCT04517396	PPAR-alpha activator	Raise HDL levels
**Atorvastatin**	NCT04801940	HMG-CoA reductase inhibitors	Raise HDL levels
	NCT04631536		
	NCT04486508		
	NCT04466241		
	NCT04380402		
**Rosuvastatin**	NCT04472611 NCT04359095	HMG-CoA reductase inhibitors	Raise HDL levels
**Simvastatin**	NCT04348695 NCT04343001	HMG-CoA reductase inhibitors	Raise HDL levels

Abbreviations: HDL, high-density lipoprotein; COVID-19, Coronavirus disease 2019; HMG-CoA, 3-hydroxy-3-methylglutaryl coenzyme A; CETP, cholesteryl ester transfer protein; PPAR, peroxisome proliferator-activated receptor; FA, fatty acids.

## Conclusion

COVID-19 has spread globally and caused significant morbidity and mortality. Patients with severe COVID-19 are characterized by a dysregulated immune response and abnormal coagulation function, which results in organ dysfunction and ultimately death. HDL possesses several well-documented functions, including regulating immune response, neutralizing endotoxins, anti-oxidation, anti-apoptosis, and anti-thrombosis formation. Multiple studies showed that HDL level, composition and functions were greatly changed in COVID-19 and lower HDL level was correlated with higher risks of severity and mortality, indicating that high HDL levels might be beneficial in COVID-19. HDL level-raising pharmacological compounds such as CETP inhibitors and fibrates are considered to be potential treatments for patients with COVID-19, and they are already in the preclinical research stage. Until now, there are still relatively few studies on the mechanisms about the protective role of HDL in COVID-19. Notably, many studies related to sepsis support that increasing the levels of HDL in septic patients may be a feasible treatment target. However, simply increasing the level of HDL does not seem to be enough to restore the function of HDL. Therefore, we still need to comprehensively understand the mechanism of HDL action in COVID-19 and improve new strategies for the treatment of patients with COVID-19, by further in-depth study on the composition, structure, and function of HDL in COVID-19.
